# Endoscopic Excision of Frontal Recess Osteoma in a Patient With Nasal Polyposis

**DOI:** 10.7759/cureus.28362

**Published:** 2022-08-24

**Authors:** Erica Gima, Shiraz Qamil, Azuin Izzati, Fadzilah Ismail, Norasnieda Md Shukri

**Affiliations:** 1 Otorhinolaryngology - Head and Neck Surgery, School of Medical Sciences, Universiti Sains Malaysia, Kubang Kerian, MYS; 2 Otorhinolaryngology, Hospital Sungai Buloh, Sungai Buloh, MYS

**Keywords:** asymptomatic presentation, functional endoscopic sinus surgery, nasal polyp, osteoma, paranasal sinuses

## Abstract

Paranasal sinus (PNS) osteomas are benign growths that are usually asymptomatic and often discovered incidentally upon imaging. Nasal polyps, on the other hand, are relatively commoner than osteomas. With the adoption of endoscopic technology in the otorhinolaryngology (ORL) field, surgeons are shifting toward less invasive surgical methods in managing such cases. We present a case of a 23-year-old male who had chronic rhinosinusitis with nasal polyps. A computed tomography (CT) done as part of his preoperative planning revealed findings suggestive of nasal polyps with left frontal recess osteoma. Both pathologies were removed successfully via an endoscopic endonasal approach.

## Introduction

Osteomas are the most common benign tumors of the paranasal sinuses (PNS), with an incidence between 3% and 6.4%. As they are slow-growing, patients are usually asymptomatic, and osteomas are often incidental findings on radiological imaging. The patient usually complains of headaches or facial pain when symptoms are present. In the past, the location of this patient’s osteoma would necessitate an external approach, but with advancements in endoscopes and endoscopic sinus surgery, we manage both lesions in a less invasive manner, sparing him any cosmetic facial defect.

## Case presentation

**Figure 1:**
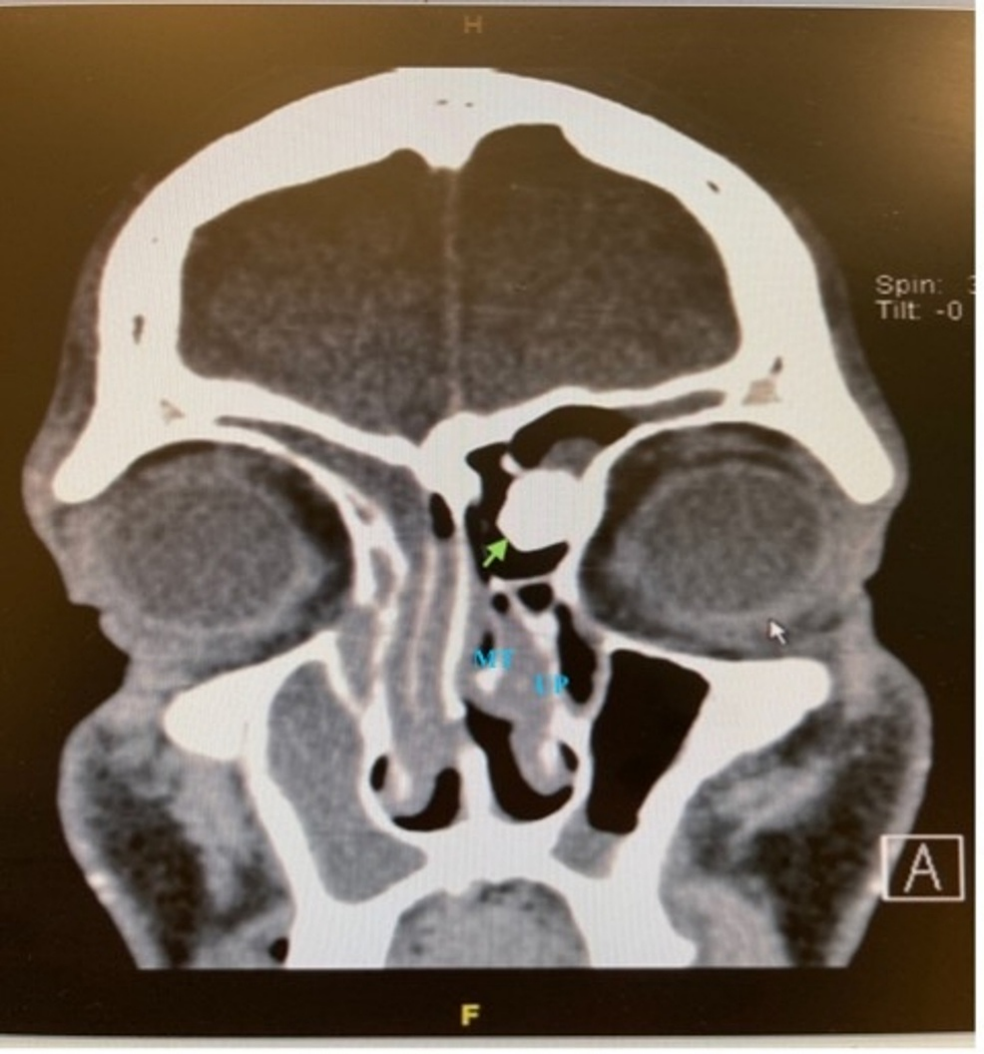
Coronal view of the patient’s CT PNS The osteoma is indicated by the green arrow. MT: middle turbinate; UP: uncinate process; CT: computed tomography; PNS: paranasal sinus

**Figure 2:**
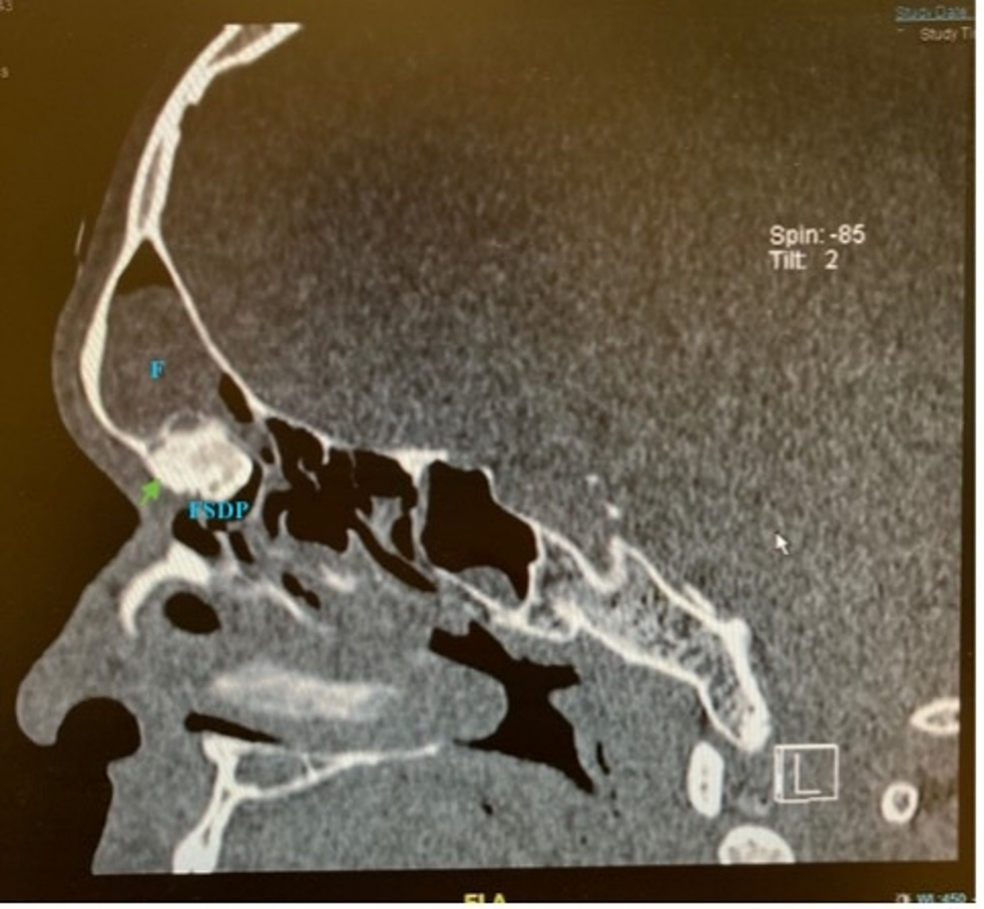
Sagittal view of the patient’s CT PNS The osteoma is indicated by the green arrow. FS: frontal sinus; FSDP: frontal sinus drainage pathway; CT: computed tomography; PNS: paranasal sinus

**Figure 3:**
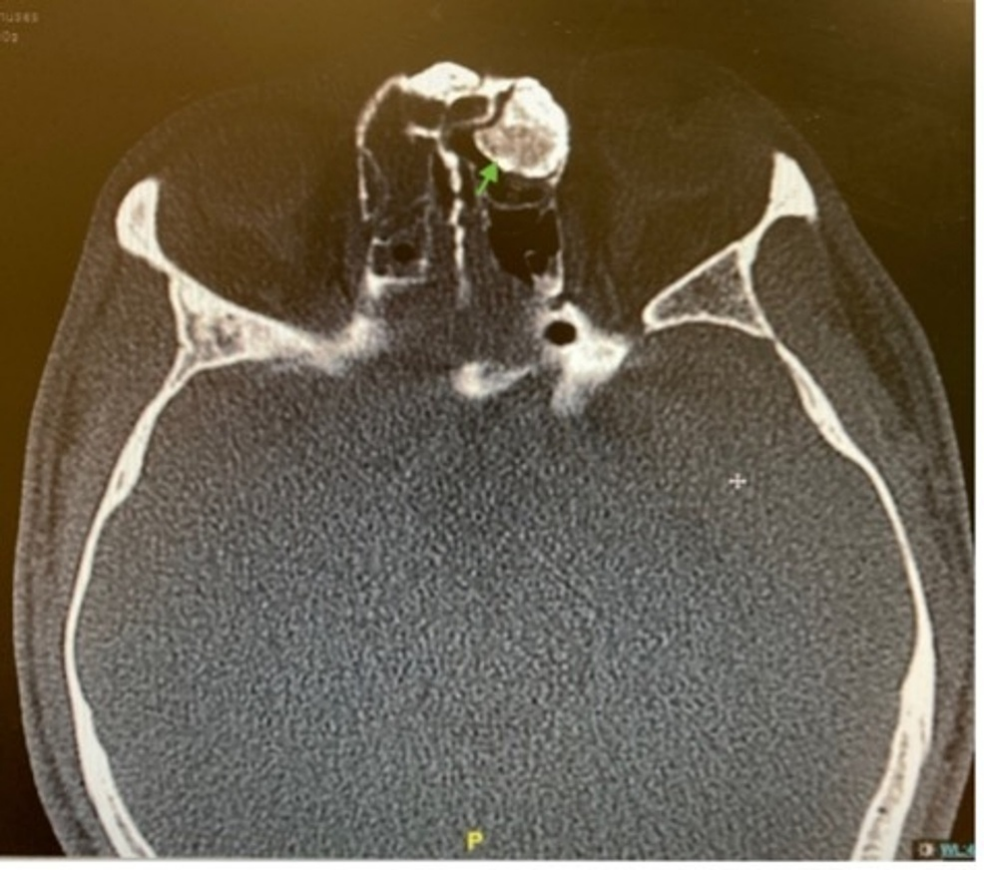
Axial view of the patient’s CT PNS The osteoma is indicated by the green arrow. CT: computed tomography; PNS: paranasal sinus

A 23-year-old male presented to our otorhinolaryngology (ORL) clinic complaining of worsening bilateral nasal blockage associated with intermittent frontal headache and midfacial pain for the past three months. He had nasal congestion and rhinitis for years but never sought medical treatment. He is a smoker, denying allergies or a history of trauma. A rigid endoscopy revealed grade 3 nasal polyps on the right and grade 2 on the left. The patient was initially treated with medical polypectomy, consisting of a two-week course of 25 mg once daily (OD) prednisolone, tablet clarithromycin 500 mg OD for two weeks and 250 mg OD for four weeks, and high-dose intranasal mometasone furoate spray. Later, he was started on regular intranasal mometasone furoate spray and tablet loratadine 10 mg OD. A computed tomography (CT) scan of his paranasal sinus (PNS) showed an osteoma at the left frontal recess measuring 1.7 × 1.5 × 1.5 cm, causing narrowing of the left frontal recess opening (Figures 1-3). There was also soft tissue density in bilateral maxillary, ethmoid, sphenoid, and frontal sinuses, which was worse on the right side.

We proceeded with functional endoscopic surgery and excision of osteoma. Intraoperatively, the polyps were removed using a microdebrider. The frontal recess was approached via Carolyn’s window. This approach gives a comprehensive view and access to the frontal recess. The osteoma was visualized at the left anterior part of the frontal recess, obscuring about 50% of the frontal recess. We drilled around the osteoma with a diamond burr to reduce its size and removed it en bloc. Anterior nasal packing was done and removed 48 hours postoperatively. The patient was discharged well on day 2 postsurgery. The histopathology examination reported inflammatory polyps and compact bone.

## Discussion

Osteomas in the PNS occur most frequently in the ethmoid sinus, followed by the frontal sinus, and rarely in the maxillary and sphenoid sinus [1]. These osteogenic tumors may occur in all age groups, with a higher incidence in those aged 40 years old and above, and they are more common in men. Due to their slow growth, patients are usually asymptomatic until they grow sufficiently large to cause compressive or obstructive symptoms. Signs and symptoms depend on the location and direction of growth of the osteoma. Ethmoid osteomas manifest earlier than frontal osteomas as the small space in ethmoid air cells increases the likelihood of extension to neighboring structures. Leading theories regarding osteoma formation include developmental, traumatic, and infective theories [2]. The developmental theory suggests that osteomas arise from embryologic remnants at junctions between membranous and endochondral ossification areas. The traumatic theory suggests that osteoblastic activity may be triggered by past injuries, whereas the infectious theory postulates that inflammatory states lead to osteoma formation. The role of genetic inheritance can be seen in Gardner’s syndrome, an autosomal dominant disease characterized by multiple osteomas, colorectal polyps, and impacted supernumerary teeth [3]. In this patient, chronic inflammation of his nasal mucosa led to the formation of nasal polyps. We postulate that the osteoma might have formed in response to an inflammatory state and infection.

Small osteomas that are asymptomatic may be managed conservatively via watchful waiting. Surgical approaches may be categorized into external, endoscopic, or a combination of both when indicated. Open approaches such as osteoplastic flap, lateral rhinotomy, external frontoethmoidectomy, and Caldwell-Luc procedure have the advantage of providing direct access at the expense of cosmesis, shorter recovery, and shorter hospital stay. Reasons cited for using open approaches include intracranial extension, intraorbital involvement, giant osteomas, and small anteroposterior diameter of the frontal sinus [4]. With time, there has been a shift toward endoscopic procedures made possible by angled instruments, drills, and navigation systems. An osteoma can be addressed by middle meatal antrostomy or Draf surgery, depending on its location. Specifically for frontal sinus osteomas, a grading system was revised by Chiu et al. [5] to guide the surgical approach. He suggested external approaches for tumors with superior or anterior attachment in the frontal sinuses or tumors extending laterally to the lamina papyracea and tumors occupying the frontal sinus (grade III and grade IV, respectively). However, with evolving techniques, the endoscopic approach’s boundaries are constantly pushed as several authors have demonstrated success in endoscopic excision of grade III and grade IV osteomas [6,7]. Although they proposed a new classification system to guide the treatment approach, Watley et al. [8] emphasized that the criteria are not absolute and that decisions should be tailored to each patient while considering the surgeon’s experience. Combined approaches may be used in tumors with extensive attachment to the frontal sinus floor or laterally extending tumors in patients with narrow interorbital distance, limiting the working space [8].

This patient’s osteoma was anteromedially located, and he had an adequate anteroposterior diameter of the frontal recess, which made it favorable for us to do a purely endoscopic excision. We used Carolyn’s window, a technique demonstrated by Richard Harvey (https://www.youtube.com/watch?v=SJgdj88iW-Q) to gain access to the frontal sinus. An inferiorly based flap was created by making a vertical incision from the axilla up to the roof of the nasal cavity, bringing it forward to the pyriform aperture and then downward along the edge of the middle turbinate. The axilla and agger nasi were drilled down with a diamond burr, giving wide access to the frontal recess, thus enabling the use of a 0° scope and straight instruments. Due to the proximity of the base of the skull and the anterior ethmoidal artery to the frontal recess, the preoperative CT scan should be reviewed thoroughly to avoid accidentally injuring these structures.

Because of its location on this patient’s anterior wall of the frontal recess, an external approach via the Lynch-Howarth incision would have given us direct access to the tumor. However, due to favorable patient factors and the surgeon’s experience, we proceeded with an endoscopic excision, resulting in a shorter hospital stay, no visible scars, and an uneventful recovery. The prognosis of the endoscopic approach is good, and recurrence is rare. When recurrence occurs, it has been attributed to the regrowth of incompletely resected osteomas [9].

## Conclusions

​Osteomas are slow-growing benign tumors that should be addressed surgically when they produce symptoms. Surgeons should perform their due diligence when deciding on the treatment approach, considering the patients’ anatomical variations and the surgeons’ capabilities. While the open approach was more often employed in the past, recent technological advancements and evolving skills have made the endoscopic approach more feasible and with a good treatment outcome.
